# Wnt3a Facilitates SARS-CoV-2 Pseudovirus Entry into Cells

**DOI:** 10.3390/ijms25010217

**Published:** 2023-12-22

**Authors:** Ivonne Melano, Hui-Jye Chen, Loveness Ngwira, Pang-Hung Hsu, Li-Lan Kuo, Lloyd Noriega, Wen-Chi Su

**Affiliations:** 1 Graduate Institute of Biomedical Sciences, China Medical University, Taichung 404, Taiwan; ivonnemelano@yahoo.com (I.M.); huijyechen@mail.cmu.edu.tw (H.-J.C.); ab801017@gmail.com (L.-L.K.); lloydnoriega@gmail.com (L.N.); 2 International Master’s Program of Biomedical Sciences, China Medical University, Taichung 404, Taiwan; tiongengwira96@gmail.com; 3 Department of Bioscience and Biotechnology, National Taiwan Ocean University, Keelung 202, Taiwan; phsu@ntou.edu.tw; 4 Center of Excellence for the Oceans, National Taiwan Ocean University, Keelung 202, Taiwan; 5 Institute of Biochemistry and Molecular Biology, National Yang Ming Chiao Tung University, Taipei 112, Taiwan; 6 Department of Medical Research, China Medical University Hospital, Taichung 404, Taiwan; 7 Drug Development Center, China Medical University, Taichung 404, Taiwan

**Keywords:** SARS-CoV-2, Wnt3a, β-catenin, canonical pathway, host factor, virus entry, ACE2

## Abstract

How ACE2 functions as the major host receptor of SARS-CoV-2 despite having low expression in the lungs is still unknown. To facilitate the development of therapeutic strategies against coronaviruses, gaining a deeper comprehension of the molecular mechanism of SARS-CoV-2 infection is imperative. In our previous study, we identified several potential host factors of SARS-CoV-2 using an shRNA arrayed screen, one of which was Wnt3a. Here, we validated the significance of Wnt3a, a potent activator of the Wnt/β-catenin signaling pathway, for SARS-CoV-2 entry into cells by evaluating the effects of its knockdown and overexpression on SARS-CoV-2 pseudotyped virus entry. Further analysis revealed that SARS-CoV-2 pseudotyped virus infection activates the canonical Wnt/β-catenin signaling pathway, which we found could subsequently stimulate ACE2 transcription. Collectively, our study identified Wnt3a as an important host factor that facilitates ACE2-mediated virus infection. Insight into the virus entry mechanism is impactful as it will aid in developing novel therapeutic strategies against current and future coronavirus pandemics.

## 1. Introduction

The COVID-19 pandemic, which has emerged as a major global health crisis, is caused by SARS-CoV-2, a positive single-stranded RNA virus belonging to the genus Betacoronavirus in the family Coronaviridae. The current vaccines primarily focus on training the immune system to recognize its Spike protein, which plays a key role in virus entry by binding to the angiotensin-converting enzyme 2 (ACE2), the primary host cell surface receptor for SARS-CoV-2. The bound SARS-CoV-2 proceeds to penetrate the cytoplasm via the cell surface or endocytic pathways. In the cell surface pathway, the Spike protein is primed by the cellular proteases furin and TMPRSS2 into S1 receptor-binding and S2 fusion-binding subunits through proteolytic cleavage at the S1/S2 and the S2′ cleavage sites, respectively [1]. After cleavage, S2 is exposed and causes the fusion of the viral and host cellular membranes, which permits the direct release of the viral contents into the cytoplasm [2]. In the endocytic pathway, SARS-CoV-2 is endocytosed into the cytoplasm and employs the cysteine peptidase cathepsin L in the endosomes to process its Spike protein to fuse with the endosomal membrane and release its viral RNA into the cytoplasm [3,4]. While ACE2 is a major receptor for SARS-CoV-2 infection [2], it is not highly expressed in the lungs, the primary organ infected by SARS-CoV-2 [5,6], suggesting that SARS-CoV-2 utilizes other host factors besides ACE2. Intending to discover druggable host factors, we performed an shRNA screen in human lung adenocarcinoma H1650 and human embryonic kidney 293T (HEK293T) cells and identified Wnt3a as one of the candidates involved in SARS-CoV-2 Spike-mediated infection [7].

Wnt3a, a member of the Wnt family of secreted glycoproteins, mediates critical signal transduction pathways in various cellular processes, including cell proliferation, apoptosis, motility, differentiation, and polarity. Two distinct pathways are believed to be activated by Wnt ligands: canonical (β-catenin-dependent) and non-canonical (β-catenin-independent) pathways [8,9]. Wnt3a is a potent activator of the canonical pathway. In the absence of Wnt ligands, the central player of the canonical pathway, β-catenin, is controlled by a destruction complex, which is comprised of adenomatous polyposis coli (APC), axis inhibition protein (Axin), casein kinase 1 (CK1), and glycogen synthase kinase 3β (GSK3β). Kinases CK1 and GSK3β phosphorylate β-catenin that leads to its ubiquitination by beta-transducin repeat-containing E3 ubiquitin protein ligase (β-TrCP), eventually resulting in proteasomal degradation. In contrast, the engagement of Wnt ligands with cell surface receptors and co-receptors, such as Frizzled (FZD) and low-density lipoprotein-related proteins (LRP5, LRP6), recruits the Dishevelled (DVL) protein together with the destruction complex, thereby preventing β-catenin degradation. Stabilized β-catenin subsequently translocates to the nucleus and acts as a transcriptional co-activator of T-cell factor/lymphoid enhancer factor (TCF/LEF) transcription factors [8,9].

Wnt3a has been studied in several viruses, such as influenza virus, Rift Valley Fever virus (RVFV) and pseudorabies virus [10,11,12,13,14,15,16]. However, conflicting studies made the involvement of Wnt in SARS-CoV-2 infection inconclusive [17,18]. Chatterjee et al. showed that silencing β-catenin expression and inhibiting its activity reduced virus infection [17], whereas Koval et al. demonstrated that the addition of several Wnt inhibitors has no effect on SARS-CoV-2 infection [18]. In this study, we validated its importance and elucidated its role in SARS-CoV-2 infection. We also demonstrated the activation of the canonical pathway by SARS-CoV-2 infection. Further experiments revealed that ACE2 transcription can be regulated by the canonical pathway. This study provided novel insights into the molecular mechanisms underlying SARS-CoV-2 infection.

## 2. Results

### 2.1. Wnt3a Is Involved in SARS-CoV-2 Entry

**Figure 1:**
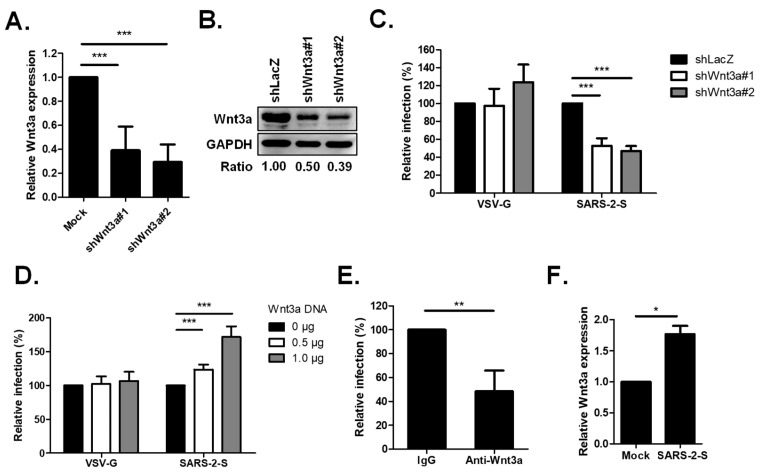
Wnt3a is important in SARS-CoV-2 entry. (**A**,**B**) Knockdown (KD) efficiency of shWnt3a in H1650 cells. (**A**) H1650 cells were infected with shRNA-carrying lentiviruses at an MOI of 3 and were selected with puromycin for 3 days. The cellular RNA of the KD cells was extracted and Wnt3a RNA expression was measured by qRT-PCR. The level of Wnt3a RNA was normalized by GAPDH RNA and compared to shLacZ. (**B**) The Wnt3a protein expressions in H1650 KD cells were analyzed by Western blotting. The ratio of band intensities was calculated using ImageJ. (**C**) KD effect of Wnt3a on Spike Vpp infection. The KD cells were transduced with either SARS-CoV-2 Spike or VSV-G Vpp, and luciferase activities were analyzed at 72 h post infection (hpi). Luciferase values were normalized by cell viability, which was detected by MTS assay, and each infection level was compared with that of the control, shLacZ. (**D**) Effect of Wnt3a overexpression on Spike Vpp infection. H1650 cells were transfected with the indicated concentrations of Wnt3a-expressing plasmid DNA. At 24 h post-transfection (hpt), the cells were infected with the Vpp and incubated for 48 h until luciferase activities were analyzed. (**E**) Anti-Wnt3a antibody competition assay on Spike Vpp infection. Pretreatment of H1650 cells with rabbit IgG or anti-Wnt3a antibody was performed for 1 h at 37 °C prior to Spike Vpp infection. Luciferase accumulations were detected at 72 hpi. (**F**) The levels of Wnt3a RNA expression in Spike Vpp-infected cells. H1650 cells were infected with Spike Vpp for 24 h at an MOI of 0.5. Wnt3a RNA expression was measured by qRT-PCR. Values represent the mean ± SD of three independent experiments. *, *p* < 0.5; **, *p* < 0.01; and ***, *p* < 0.001 compared with controls (*n* = 3).

To validate the importance of Wnt3a in SARS-CoV-2 entry, we utilized H1650 cells, the cell line we previously used for the shRNA screening assay and which has also been confirmed by others to support SARS-CoV-2 infection [7,19]. We silenced Wnt3a in H1650 cells using two unique shRNAs and subsequently infected them with SARS-CoV-2 pseudotyped particles (Vpp) harboring the Spike protein. qRT-PCR and Western blot analysis demonstrated significant reductions in Wnt3a gene expression (Figure 1A,B), which correspondingly diminished Spike Vpp infection to 53% and 47% (Figure 1C), indicating that the reduction in Wnt3a expression decreases virus entry. This finding is further supported by the overexpression of Wnt3a. H1650 cells transfected with different doses of Wnt3a plasmid showed an increase in Spike Vpp infectivity, with values of 124% and 172% (Figure 1D). Moreover, blocking Wnt3a by antibody neutralization assay using an anti-Wnt3a antibody resulted in lower Spike Vpp infectivity compared to that of IgG control cells (Figure 1E). Notably, the infection of the pantropic vesicular stomatitis virus G (VSV-G) Vpp was not altered by either the loss or overexpression of Wnt3a (Figure 1C,D), indicating the specificity of Wnt3a in SARS-CoV-2 entry. We also checked the Wnt3a expression in Spike Vpp-infected cells by qRT-PCR and found that the Wnt3a expression is markedly increased in virus-infected cells compared to mock-infected cells (Figure 1F). Altogether, these findings prove the importance of Wnt3a in SARS-CoV-2 Spike-mediated entry.

### 2.2. Loss of Wnt3a-Affected Infectivity of SARS-CoV-2 Variants

**Figure 2:**
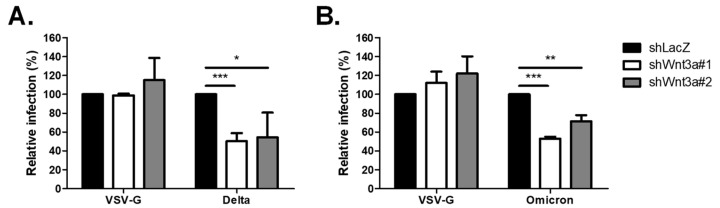
Knockdown of Wnt3a decreased infection of Spike Vpp variants. KD cells were infected with Spike Vpp variants, including Delta (**A**) and Omicron (**B**), and luciferase assay was conducted at 72 hpi. Values represent the mean ± SD of three independent experiments. *, *p* < 0.05; **, *p* < 0.01; and ***, *p* < 0.001 compared with controls (*n* = 3).

In efforts to contain the pandemic, a number of vaccines and monoclonal antibodies against SARS-CoV-2 have been authorized. However, their efficacy is challenged by the continuous emergence of new variants that harbor mutations in the Spike protein, such as Delta and Omicron [20,21,22,23]. Thus, targeting the more genetically stable host factors provides an alternative strategy for developing new antiviral therapies. To this end, we investigated whether the Delta (B.1.617.2) and Omicron (B.1.1.529) variants also employ Wnt3a for virus infection. As shown in Figure 2, silencing of Wnt3a reduced Delta Vpp infection to 50% and 55% (Figure 2A), and Omicron Vpp infection to 53% and 71% (Figure 2B). These data show that Wnt3a is involved in SARS-CoV-2 infection, affecting not only the wild-type SARS-CoV-2 but also the current variants of concern.

### 2.3. Wnt3a Inhibitors Attenuated SARS-CoV-2 Vpp Infection

**Figure 3:**
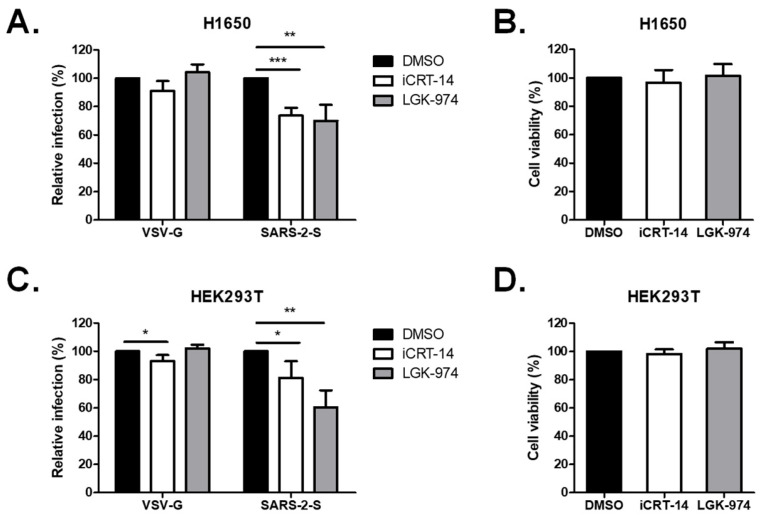
Treatment with Wnt3a inhibitors diminished SARS-CoV-2 entry in H1650 and HEK293T cells. (**A**,**B**) H1650 were pre-treated for 1 h with either 100 nM iCRT14 or 1 nM LGK-974 and were infected with Vpp overnight in the presence of the inhibitors. (**C**,**D**) HEK293T cells were pre-treated for 1 h with either 10 nM iCRT14 or 0.01 nM LGK-974 and were infected with Vpp overnight in the presence of the inhibitors. (**A**,**C**) Rates of virus infection and (**B**,**D**) cell viability were analyzed at 72 hpi by luciferase assay and MTS assay, respectively. Values represent the mean ± SD of three independent experiments. *, *p* < 0.05; **, *p* < 0.01; and ***, *p* < 0.001 compared with controls (*n* = 3).

Several inhibitors against Wnt signaling have been developed for research use. We infected the H1650 cells in the presence of iCRT14, a potent Wnt/β-catenin inhibitory drug that blocks the direct interaction of β-catenin and TCF/LEF transcription factors, and LGK-974, a porcupine inhibitor that blocks the secretion of Wnt3a. Treatment with iCRT-14 and LGK-974 significantly decreased Spike Vpp infectivity to 74% and 70%, respectively, but not VSV-G infectivity, at non-cytotoxic doses (Figure 3A,B). We further repeated the experiments in HEK293T cells. Similarly, treatment with iCRT-14 and LGK-974 reduced Spike Vpp infectivity in HEK293T cells to 81% and 60%, respectively, at non-cytotoxic doses (Figure 3C,D). Taken together, these data confirm the importance of Wnt3a in SARS-CoV-2 entry.

### 2.4. Activation of the Wnt/β-Catenin Signaling Pathway by SARS-CoV-2 Infection

**Figure 4:**
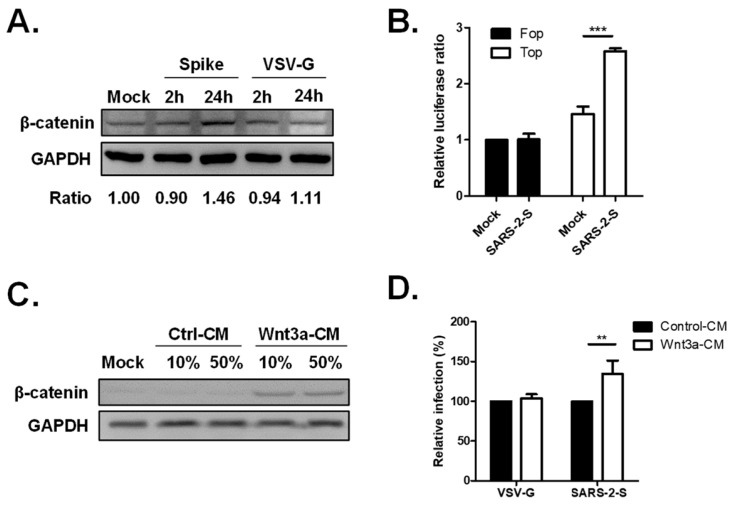
The Wnt/β-catenin signaling pathway is activated in SARS-CoV-2 Spike Vpp infection. (**A**) β-catenin expression in Spike Vpp-infected cells. 293T cells were infected with Spike or VSV-G Vpp at MOI 0.5 for 2 and 24 hpi. Cellular lysates were subjected to Western blot analysis using mouse anti-β-catenin and rabbit anti-GAPDH antibodies. The ratio of the band intensities was calculated using ImageJ. (**B**) β-catenin activity in Spike Vpp-infected cells. HEK293T-ACE2 cells were transfected with TOPFlash or FOPflash reporters together with pTK-Rluc normalization vector. At 24 hpt, the cells were infected with SARS-CoV-2-Spike GFP Vpp for 24 h. Firefly luciferase activities were normalized to *Renilla* luciferase activities and are shown as fold change relative to mock-infected cells. (**C**) Validation of Wnt3a-conditioned media (CM). H1650-ACE2 cells were pre-treated for 24 h with the indicated concentrations of the control or Wnt3a-CM and were harvested for Western blot analysis. (**D**) Spike Vpp infection in Wnt3a-CM-treated cells. H1650 cells were incubated with control or Wnt3a-CM for 24 h and subsequently infected with Spike Vpp for 24 h. Luciferase activities are shown as fold change in Wnt3a-CM-treated cells relative to control CM-treated cells. Values represent the mean ± SD of three independent experiments. **, *p* < 0.01; and ***, *p* < 0.001 compared with controls (*n* = 3).

Since the disruption of Wnt/β-catenin signaling by iCRT-14 treatment diminished SARS-CoV-2 Spike Vpp infection, we were interested in further elucidating the role of Wnt/β-catenin signaling in SARS-CoV-2 infection. We checked the β-catenin expression of Spike-Vpp infected H1650 cells by Western blot analysis and found that the β-catenin expression is stronger in Spike Vpp-infected cells at 24 h post infection (hpi) compared to mock-infected cells (Figure 4A). Although β-catenin expression also increased upon VSV-G infection at 24 hpi, it remained lower than that observed in Spike Vpp. These data imply that the Wnt/β-catenin pathway is activated upon Spike Vpp infection. To gain more insight into the β-catenin signaling activation induced by SARS-CoV-2 infection, we used the TCF/LEF-1-luciferase reporter to determine whether SARS-CoV-2 infection affects β-catenin activity. H1650 cells were transfected with either TOPflash, which contains TCF binding sites, or FOPflash containing mutated binding sites as a control. Subsequently, the cells were infected the next day with Spike Vpp for 24 h until luciferase assay was performed to detect TOPflash and FOPflash activities. Spike Vpp infection resulted in a significant increase in TOPflash activity compared to mock-infected samples, but no increase was detected in FOPflash activities (Figure 4B), proving the activation of the β-catenin-mediated transcription upon Spike Vpp infection. It has been demonstrated that the knockdown of β-catenin reduced SARS-CoV-2 infection [17], hence, to further investigate the relationship between β-catenin and SARS-CoV-2 infection, we employed Wnt3A-conditioned medium (CM). As shown in Figure 4C, higher β-catenin protein levels were detected in Wnt3a-CM-treated cells compared to those in control-CM-treated cells, indicating that Wnt3a-CM effectively activates the Wnt3a/β-catenin signaling pathway. Next, we incubated the cells with Wnt3a-CM prior to infection. Wnt3a-CM treatment significantly enhanced Spike Vpp infectivity to 135% compared to cells treated with control CM, whereas the infectivity of VSV-G remained unaffected by Wnt3a-CM treatment (Figure 4D), thus showing that the activation of the β-catenin signaling pathway benefits SARS-CoV-2 entry.

**Figure 5:**
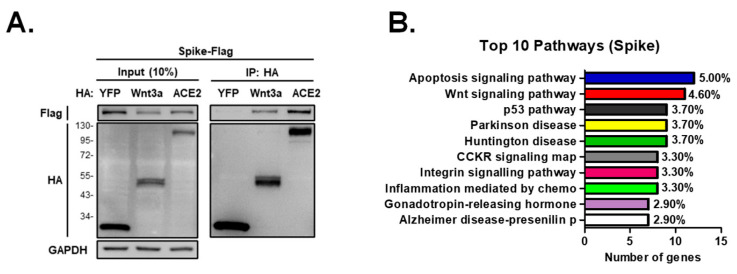
SARS-CoV-2 Spike is associated with Wnt signaling pathway. (**A**) Immunoprecipitation analysis of Spike and Wnt3a. 293T cells were transfected with flag-tagged Spike together with HA-tagged YFP, Wnt3a, or ACE2 proteins. Cell lysates were harvested at 48 hpt, incubated with HA agarose beads overnight, and subjected to Western blotting using rabbit anti-flag and anti-HA antibodies. (**B**) Pathways activated by Spike protein. 293T cells were transfected with Spike proteins and were subjected to immunoprecipitation-mass spectrometry. The number of assigned genes and the percentage of gene frequency in the top 10 pathways detected by Panther analysis are shown (http://www.pantherdb.org; accessed on 10 August 2022).

We were next curious how SARS-CoV-2 activates the β-catenin signaling pathway; hence, we conducted immunoprecipitation analysis and found that Wnt3a complexes with the Spike protein (Figure 5A). We then performed immunoprecipitation coupled with mass spectrometry to identify the proteins that complex with Spike. The detected proteins were functionally clustered using the Panther pathway classification system. Analysis shows that the Wnt signaling pathway was one of the most represented pathways among the proteins that complex with Spike (Figure 5B). These data collectively imply that the SARS-CoV-2 Spike protein stimulates the Wnt/β-catenin pathway to aid virus entry.

### 2.5. The Wnt3a/β-Catenin Pathway Regulates ACE2 Transcription

**Figure 6:**
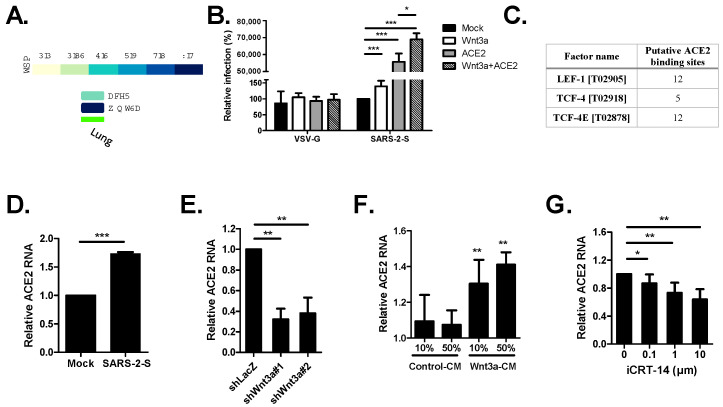
ACE2 transcription is regulated by the Wnt3a/β-catenin pathway. (**A**) Tissue transcriptional expression of ACE2 and Wnt3a in the lungs from the Genotype-Tissue Expression (GTEx) Project. TPM, transcripts per million. (**B**) Spike Vpp infection in Wnt3a and ACE2-transfected cells. H1650 cells transfected with the indicated plasmids were transduced with Spike or VSV-G Vpp at 24 hpt, and luciferase accumulations were measured at 48 hpi. (**C**) Summary of computer-assisted search for the putative binding sites of selected transcription factors (LEF-1, TCF-4, and TCF-4E) in the promoter regions of human ACE2 using Alggen Promo software, V 3.0.2. (http://alggen.lsi.upc.es; accessed on 14 September 2022). Factor name—transcription factor with its TRANSFAC (V 8.3) database accession number. Putative binding sites—number of predicted binding sites. (**D**) ACE2 RNA expression in Spike Vpp-infected cells. ACE2 RNA expression was measured in Spike Vpp-infected cells (MOI 0.5) at 24 hpi by qRT-PCR and compared to mock-infected cells. (**E**) ACE2 RNA levels in Wnt3a KD cells. ACE2 RNA expression was measured in Wnt3a KD cells by qRT-PCR and compared to shLacZ. (**F**) ACE2 RNA levels in Wnt3a-CM-treated cells. ACE2 RNA levels were measured in H1650 cells treated with the indicated concentrations of control or Wnt3a-CM for 24 h. (**G**) Effect of disruption of β-catenin pathway on ACE2 RNA levels. H1650 cells were treated with the indicated concentrations of iCRT14 for 24 h and were subsequently harvested for qRT-PCR. The levels of ACE2 RNA were normalized by GAPDH RNA. Values represent the mean ± SD of three independent experiments. *, *p* < 0.05; **, *p* < 0.01; and ***, *p* < 0.001 compared with controls (*n* = 3).

Based on the GTEx portal, Wnt3a expression in the lungs is higher than ACE2 expression (Figure 6A), hinting that Wnt3a may be used to compensate for low ACE2 expression upon virus infection. To probe the relationship between Wnt3a and ACE2 for SARS-CoV-2 infection, we co-transfected Wnt3a and ACE2 plasmids into H1650 cells and subsequently infected the cells with Vpp. We discovered significantly higher Spike Vpp infectivity in Wnt3a+ACE2 cells (68,953%) compared to individual expression of Wnt3a (139%) and ACE2 (55,650%) (Figure 6B). This suggests that Wnt3a contributes to ACE2-mediated virus infection. Since β-catenin binds to TCF/LEF transcription factors to initiate the transcription of target genes, we were curious whether Wnt3a aids ACE2 by regulating its transcription. Using ALGGEN PROMO software version 3.0.2 to predict putative transcription binding sites for LEF-1, TCF-4, and TCF-4E [24,25], we detected several putative binding sites on the promoter region of ACE2 (Figure 6C). Moreover, we found a significant increase in ACE2 RNA expression upon Spike Vpp infection, suggesting an enhancement of ACE2 transcription (Figure 6D). To validate whether Wnt3a is linked with the gene transcription of ACE2, the total RNA of Wnt3a knockdown cells was harvested and analyzed by qRT-PCR to quantify ACE2 gene expression. Significantly lesser ACE2 expression was detected in Wnt3a KD cells (Figure 6E), showing that the manipulation of Wnt3a expression affects ACE2 transcription. To investigate whether the Wnt3a/β-catenin pathway activates ACE2 transcription, H1650 cells were incubated with Wnt3a-CM to stimulate the β-catenin pathway. Wnt3a-CM-treated cells, but not the control-CM-treated cells, demonstrate significantly higher ACE2 RNA expression compared to mock cells (Figure 6F), suggesting the activation of ACE2 transcription. Furthermore, we used iCRT14 to disrupt the interaction of β-catenin and TCF/LEF transcription factors. This resulted in a dose-dependent decrease in ACE2 expression (Figure 6G). Overall, the data infer that the Wnt3a/β-catenin pathway mediates ACE2 transcription.

## 3. Discussion

The Wnt/β-catenin signaling pathway has been proven to be involved in the life cycle of several viruses. In the context of SARS-CoV-2, there have been conflicting studies about the role of Wnt in SARS-CoV-2 infection. Chatterjee et al. were the first to demonstrate the role of β-catenin in SARS-CoV-2 infection by iCRT-14 treatment and β-catenin silencing in Vero cells, which have both effectively reduced virus infection [17]. On the other hand, Koval et al. had tested several Wnt inhibitors, namely clofazimine, MU17, F2-99 and ICG-001, in Calu-3 and A549-ACE2-TMPRSS2 cells and have shown no significant inhibition in SARS-CoV-2 infection, hence contradicting the initial observations of the possible role of the canonical signaling pathway for SARS-CoV-2 infection [18]. Nonetheless, our findings agree with those of Chatterjee et al. that the canonical Wnt signaling pathway is important for SARS-CoV-2 infection. We discovered that the upstream component of the pathway, Wnt3a, is important for SARS-CoV-2 infection and inhibition of Wnt3a secretion by the treatment of LGK-974 significantly reduced virus infection. Since SARS-CoV-2 Spike protein interacts with Wnt3a, we propose that SARS-CoV-2 associates with Wnt3a to activate the Wnt/β-catenin signaling pathway, which can subsequently stimulate the transcription of ACE2, the major receptor of SARS-CoV-2. Nevertheless, further analysis is still necessary to provide mechanistic insights on the functional interplay between Wnt3a and SARS-CoV-2 entry.

Furthermore, our study demonstrates that Wnt3a is involved in the infection of not only wild-type SARS-CoV-2, but also Delta and Omicron variants. Silencing of Wnt3a reduced the infectivity of both Delta and Omicron variants, highlighting the potential of targeting Wnt3a as a strategy to combat different SARS-CoV-2 variants. This proves the effectiveness of targeting host factors, which are genetically stable, against virus variants. The ideal antiviral agent would be potently active against both current and future variants. Since the host is more genetically stable than viruses, one approach is to develop host-directed antivirals (HDAs) that inhibit human host cell proteins responsible for viral infection and replication [26]. Several HDAs have been approved as effective treatments against viral infections [27]. For instance, Maraviroc, a C-C chemokine receptor type 5 (CCR5 or R5) antagonist, has been approved for clinical use in treating R5-tropic human immunodeficiency virus (HIV)-1. By targeting host proteins, HDAs can potentially overcome the challenges posed by viral mutations and drug resistance [27]. In this study, we utilized two Wnt/β-catenin signaling pathway inhibitors, iCRT-14 and LGK-974, which significantly reduced Spike Vpp infectivity. These results further support the potential of Wnt3a inhibitors as antiviral agents against SARS-CoV-2. Several compounds targeting the Wnt/β-catenin signaling pathway are already in clinical trials for cancer therapy [28]. LGK974, also known as WNT974, is among the compounds currently undergoing phase I clinical trials in patients with advanced solid tumors [29]. These compounds have the potential to be repurposed as HDAs against SARS-CoV-2 infection.

Interestingly, our results indicate that Wnt3a may regulate ACE2 transcription. Our findings suggest that the Wnt3a/β-catenin pathway mediates ACE2 transcription and may contribute to ACE2-mediated virus infection. Notably, a higher ACE2 protein level was detected in the lungs of severe COVID-19 patients [30]. Though we identified putative binding sites for Wnt/β-catenin pathway transcription factors within the ACE2 promoter region, further study, such as chromatin-level analysis, is needed to validate these binding sites and explore the Wnt3a/β-catenin pathway-regulated transcription of ACE2.

Several factors, such as older age, diabetes and obesity, have been considered to be predictors of COVID-19 severity [31]. Interestingly, Wnt5a and Wnt11 have been suggested to be potential biomarkers of COVID-19 severity, with high levels of Wnt5a correlated to poor prognosis and increased Wnt11 expression indicating efficient inhibition of inflammatory responses caused by SARS-CoV-2 infection [32]. Our data demonstrate an increase in Wnt3a RNA levels upon SARS-CoV-2 infection and show that inhibition of Wnt3a secretion by the treatment with LGK-974 reduces SARS-CoV-2 entry. It is plausible that Wnt3a levels may also be considered as a biomarker of enhanced virus infection; however, extensive investigation on the dynamic regulation of Wnt3a expression during viral infection using patient plasma is necessary.

This study has several limitations. First, the experiments were primarily conducted using H1650 and HEK293T cell lines. While these cell lines are commonly used in research, the findings may not fully represent the complexity of SARS-CoV-2 infection in primary human cells or other relevant cell types. Second, we utilized Vpp instead of authentic SARS-CoV-2 virus. Vpp only harbors the SARS-CoV-2 Spike protein, but lacks the other structural viral proteins—Envelope, Membrane and Nucleocapsid proteins, which can also play important roles on cellular pathways. However, while the behavior of the Vpp may not fully recapitulate the complexities of authentic viral infection, including viral replication and spread, Vpp provides a useful model for studying viral entry [33]. We have previously demonstrated that the known receptors for SARS-CoV-2 (i.e., ACE2, cathepsin L and TMPRSS2) were detected in our screening assay using SARS-CoV-2 Spike Vpp. Furthermore, disruption of endocytosis by the addition of inhibitors of endosomal acidification (i.e., ammonium chloride and bafilomycin A1) led to decreased Vpp entry, demonstrating that our SARS-CoV-2 Spike Vpp enters the cells through the endocytosis pathway, similarly to the live virus [7]. Moreover, our findings on the inhibiting effect of iCRT14 treatment on Spike Vpp infection were also observed in the authentic virus [17]. Third, our study is primarily based on in vitro experiments, and the findings need to be further validated and expanded upon in animal models and clinical studies to determine the translational potential of targeting Wnt3a as an antiviral therapy for SARS-CoV-2.

## 4. Materials and Methods

### 4.1. Cell Culture

H1650 and HEK293T cells were cultured in RPMI (Gibco, Paisley, UK) and Dulbecco’s modified Eagle’s medium (DMEM) (Gibco, Grand Island, NY, USA), respectively, supplemented with 10% fetal bovine serum (FBS) (Cytiva, Marlborough, MA, USA) and antibiotics (100 U/mL penicillin G and 100 μg/mL streptomycin). Generation of stable Wnt3a knockdown H1650 cells was performed by lentivirus transduction ofpLKO.1-shWnt3a. Stable cells were selected using 3 μg/mL puromycin. Establishment of H1650 and HEK293T cells stably expressing human ACE2 (H1650-ACE2 and HEK293T-ACE2) was conducted as previously described [7]. All the cells were cultured under 5% CO_2_ at 37 °C.

### 4.2. Plasmids and Viruses

The pLKO.1-shRNA vectors used were TRCN0000072240 (shLacZ), TRCN0000244630 (shWnt3a#1), and TRCN0000244631 (shWnt3a#2). pcDNA(TM)3.1(+)-2019-nCoV-S (Wuhan strain wild type, Delta B.1.617.2, and Omicron B.1.1.529) were provided by RNA Technology Platform and Gene Manipulation Core, Academia Sinica, Taiwan. The construction of pCAG.2-SARS-2-S-Flag, pCAG.2-SARS-2-S-HA, pCAG.2-ACE2-HA, pCAG.2-ACE2-Flag, pLKO-AS3w-SARS-2-S1-Flag.bsd, and pLKO-AS3w-SARS-2-S2-Flag.bsd were performed by PCR amplification as described previously [7]. The construction of pCAG.2-HA-Wnt3a-HA was performed by amplifying the desired sequences from commercially available Wnt3a cDNA (MHS6278-211689567) using PCR amplification with primers 5′-ATCTCCAGCACAGTGGCATGGCCCCACTCGGATACTTC-3′ and 5′-TCTGCTCGAGCGTGGAACCTTCCCAGCT-CGAC-3′ and inserted into BstXI and XhoI sites of pCAG.2. The constructs were verified by Sanger sequencing. The virus pseudotyped particles (Vpp) were prepared as previously described [34]. pcDNA3.1-S with truncation of C-terminal 18 amino acids (for SARS-CoV-2 spike Vpp) or pMD2.G (for VSVG Vpp), pCMV-d8.9, and pLAS3w-FLuc.puro (luciferase reporter) were co-transfected into HEK293T cells.

### 4.3. Antibodies and Reagents

Rabbit anti-Wnt3a and anti-Flag tag antibodies were purchased from Cell Signaling Technology, Danvers, MA, USA (2721s, and 2368s). Rabbit anti-GAPDH and goat anti-rabbit IgG antibodies were purchased from GeneTex (GTX100118 and GTX213110-01). Mouse anti-β-catenin was procured from BD Biosciences (BD610154). Rabbit anti-HA tag antibody was purchased from Millipore (04-902). Normal rabbit IgG was purchased from SantaCruz (sc-2027). Lipofectamine™ 2000 Transfection Reagent (Invitrogen, Carlsbad, CA, USA) and TransIT^®^-LT1 Transfection Reagent (Mirus Bio, Madison, WI, USA) were used for plasmid transfection of H1650 and HEK293T cells, respectively. iCRT-14 and LGK-974 were purchased from MedChemExpress, Monmouth Junction, NJ, USA. The control and Wnt3a-conditioned media (CM) were prepared as previously described [35].

### 4.4. RT-qPCR

TRIzol reagent (Ambion, Carlsbad, CA, USA) and M-MLV Reverse Transcriptase (Invitrogen, Carlsbad, CA, USA) were used for RNA extraction and cDNA synthesis, respectively. Quantitative PCR analysis was conducted following the standard TaqMan method with the Universal Probe Library system (Roche, Indianapolis, IN, USA) using the following primers and probes: for Wnt3A: 5′-GGAGAAGCACCGGGAGTC-3′ and 5′-GGGCACCTTGAAGTAGGTGT-3′ with Universal Probe 28; for ACE2: 5′- TGGGAGATGAAGCGAGAGAT-3′ and 5′-ATGCGGGGTCACAGTATGTT-3′ with Universal Probe 77; for GAPDH: 5′-AGCCACATCGCTCAGACAC-3′ and 5′-GCCCAATACGACCAAATCC-3′ with Universal Probe 60.

### 4.5. Western Blotting

To extract cell lysates, the M-PER™ Mammalian Protein Extraction Reagent (ThermoFisher, Rockford, IL, USA) containing 50× protease inhibitor (Roche, Mannheim, Germany) and the 4× Laemmli Sample Buffer (Bio-rad, Hercules, CA, USA) containing 2-mercaptoethanol (Aldrich, St. Louis, MO, USA) were added to the cells before boiling at 95 °C. Proteins were separated by sodium dodecyl sulfate-polyacrylamide gel electrophoresis (SDS-PAGE) and transferred to a polyvinylidene difluoride (PVDF) membrane. In addition, 5% skim milk prepared in phosphate-buffered saline with Tween 20 (PBST) was used for blocking and dilution of antibodies. The membranes were stained with the indicated primary and secondary antibodies on a shaking rotor. All antibodies were diluted in a blocking buffer. Protein bands were detected using Immobilon Western Chemiluminescent HRP Substrate (Millipore, Burlington, MA, USA) and ImageQuant LAS 4000 (GE Healthcare Life Sciences, Uppsala, Sweden).

### 4.6. Luciferase and MTS Assay

The Bright-Glo™ Luciferase Assay System (Promega, Madison, WI, USA) was used to estimate the Vpp infectivity according to the manufacturer’s instructions. Briefly, the luciferase substrate was added onto the wells. Following cell lysis for two minutes at room temperature, the luminescence of the samples was measured using the Synergy™ H4 Hybrid Microplate Reader (BioTek, Göteborg, Sweden). To normalize luminescence values and to quantify viable cells, the cells were subjected to MTS assay using 3-(4,5-dimethylthiazol-2-yl)-5-(3-carboxymethoxyphenyl)-2-(4-sulfophenyl)-2H-tetrazolium (Promega, Madison, WI, USA). Absorbance was determined at 490 nm using the microplate reader when the MTS turned brown. Values from control cells were calculated at 100% infectivity and viability.

### 4.7. Antibody Competition Assay

A rabbit anti-Wnt3a antibody or a rabbit IgG as control were added to the cells at 37 °C for 1 h. Spike Vpp was then inoculated onto the cells by centrifugation for 30 min at 1200 rpm. The cells were incubated at 37 °C for another 1 h. The media were then removed and changed with complete culture media. Luciferase assay was conducted at 72 hpi.

### 4.8. Immunoprecipitation Assay

Cell lysates harvested using M-PER containing protease inhibitor were centrifuged to collect the supernatant. Pierce™ Anti-HA Agarose (ThermoFisher, Rockford, IL, USA) was added to the supernatant and the mixture was rotated overnight at 4 °C. Following incubation, wash buffer I (50 mM Tris (pH 7.5), 150 mM NaCl, and 0.1% Triton X-100) was used to wash the immunoprecipitates three times. The samples were subsequently subjected to SDS-PAGE and Western blotting analysis.

### 4.9. Dual Luciferase Activity Assay

The Dual Luciferase^®^ Reporter Assay System (Promega, Madison, WI, USA) was performed according to manufacturer’s instruction. Briefly, the cells were detached from the plate by pipetting with PBS. After removal of PBS by centrifugation, the cells were lysed by adding the 1× passive lysis buffer for 15 min at room temperature. The firefly luciferase was detected by adding the luciferase assay substrate. Subsequent addition of Stop & Glo^®^ Substrate allowed the detection of the *Renilla* luciferase. The firefly luciferase activities were normalized by *Renilla* luciferase activity.

### 4.10. LC-MS/MS

Cell lysates (1 mg) from mock-transfected or Spike-HA-transfected HEK293T cells were immunoprecipitated with anti-HA agarose beads. The immunoprecipitates were separated by SDS-PAGE, and the gel was stained with PageBlue Protein Staining Solution (ThermoFisher, Rockford, IL, USA) to visualize the protein bands. The band of interest was excised from the gel and subjected to in-gel digestion as described previously [36]. The resulting peptides were analyzed by liquid chromatography-tandem mass spectrometry (LC-MS/MS) using an Orbitrap Fusion mass spectrometer (ThermoFisher) equipped with an EASY-nLC 1200 system (ThermoFisher) and an EASY-Spray HPLC column (75 μm × 150 mm, 3 μm, 100 Å). MS/MS spectra were acquired in a collision-induced dissociation (CID) mode with 35% normalized collision energy. The raw MS data files were converted to an mgf format using msConvert (version 3.0.18165, ProteoWizard, Palo Alto, CA, USA) and analyzed by Mascot (version 2.3, Matrix Science Inc., Boston, MA, USA) to identify peptides and proteins from MS/MS ion searches.

### 4.11. Statistics

To evaluate statistical significances, conventional Student’s *t*-test was performed. The tests were based on the results of at least three independent experiments. The data are presented as means ± standard deviations (SD). Statistically significant values were considered when *p* < 0.05 (*), *p* < 0.01 (**) and *p* < 0.001 (***).

## 5. Conclusions

Our discovery of the important role of Wnt3a in SARS-CoV-2 infection provides new insights into the understanding of virus–host interactions. Targeting Wnt3a and the Wnt/β-catenin pathway may offer new avenues for the development of antiviral therapies against SARS-CoV-2 and its variants. Further studies are warranted to fully understand the molecular mechanisms underlying the relationship between Wnt3a, the Wnt/β-catenin pathway, and SARS-CoV-2 infection, which could potentially lead to the development of novel therapeutics for COVID-19.

## Data Availability

Data are contained within the article.
